# The Enhancement of CO Oxidation Performance and Stability in SO_2_ and H_2_S Environment on Pd-Au/FeO_X_/Al_2_O_3_ Catalysts

**DOI:** 10.3390/ma16103755

**Published:** 2023-05-16

**Authors:** Qingrong He, Xuwei Wang, Yimeng Liu, Weimin Kong, Shanshan Ren, Yun Liang, Min Tang, Shuyuan Zhou, Yanchun Dong

**Affiliations:** 1 School of Light Industry and Engineering, South China University of Technology, Guangzhou 510640, China; 2 State Key Laboratory of NBC Protection for Civilian, Beijing 100083, China

**Keywords:** Pd-Au, SO_2_, H_2_S, CO oxidation, Au/FeO_x_/Al_2_O_3_

## Abstract

Carbon monoxide (CO) is a colourless, odourless, and toxic gas. Long-term exposure to high concentrations of CO causes poisoning and even death; therefore, CO removal is particularly important. Current research has focused on the efficient and rapid removal of CO via low-temperature (ambient) catalytic oxidation. Gold nanoparticles are widely used catalysts for the high-efficiency removal of high concentrations of CO at ambient temperature. However, easy poisoning and inactivation due to the presence of SO_2_ and H_2_S affect its activity and practical application. In this study, a bimetallic catalyst, Pd-Au/FeO_x_/Al_2_O_3_, with a Au:Pd ratio of 2:1 (wt%) was formed by adding Pd nanoparticles to a highly active Au/FeO_x_/Al_2_O_3_ catalyst. Its analysis and characterisation proved that it has improved catalytic activity for CO oxidation and excellent stability. A total conversion of 2500 ppm of CO at −30 °C was achieved. Furthermore, at ambient temperature and a volume space velocity of 13,000 h^−1^, 20,000 ppm CO was fully converted and maintained for 132 min. Density functional theory (DFT) calculations and in situ FTIR analysis revealed that Pd-Au/FeO_x_/Al_2_O_3_ exhibited stronger resistance to SO_2_ and H_2_S adsorption than the Au/FeO_x_/Al_2_O_3_ catalyst. This study provides a reference for the practical application of a CO catalyst with high performance and high environmental stability.

## 1. Introduction

Carbon monoxide produced from the inadequate combustion of fossil fuels in the chemical industry, with inefficient fire bursts and explosions in a sealed environment, poses significant safety risks to human life. Carbon monoxide poisoning is the most common cause of gas poisoning in the population, and there is no effective antidote. The pathophysiology of carbon monoxide poisoning includes reducing overall oxygen transmission and inhibiting mitochondrial respiration [1]. Therefore, the removal of CO via efficient catalysts is essential to ensure human health and safety.

Gold nanoparticles have been extensively used in catalytic oxidation reactions because of their excellent catalytic properties [2,3,4,5,6,7]. Supported nano-gold catalysts exhibit improved performance in the catalytic oxidation of CO at low (constant) temperatures, achieving complete conversion at high concentrations and room temperature [8,9]. Their performance is affected mainly by the size of the gold nanoparticles and their interaction with the support [10]. The preparation methods for supported nano-gold catalysts include impregnation [11], deposition–precipitation [12], coprecipitation [13], chemical vapour precipitation [14], and liquid-phase reduction [15]. Among them, the deposition–precipitation method is widely used because it is convenient to operate and can prepare particles with small sizes and uniform distributions [10]. FeO_x_ particles are a mixture of Fe_2_O_3_ and Fe_3_O_4_, which are supported on Al_2_O_3_. These are beneficial for the good dispersion of nano-gold particles. The oxygen vacancy on the supported nano-gold catalyst is the activation centre of oxygen molecules [16], and support selection plays a significant role in improving activity. α-Fe_2_O_3_ has abundant oxygen vacancies that can activate oxygen during the reaction process and is an excellent carrier for gold nanoparticles [17,18,19]. Guoyan Ma et al. [20] prepared γ-Al_2_O_3_-supported Cu, and the oxidation activity of the catalyst with Fe as an additive was significantly higher than that of pure Cu because the addition of Fe formed more oxygen vacancies and promoted the activity of the catalyst. Chunlei Wang et al. [21] reported the selective deposition of FeO_x_ coatings onto SiO_2_-supported Ir nanoparticles using atomic layer deposition (ALD) technology, which allows precise customisation of IrFeO interfaces to optimize the catalytic performance of PROX reactions. Compared with the uncoated Ir/SiO_2_ samples, the FeO_x_-coated Ir/SiO_2_ samples showed significantly enhanced activity.

Studies have also shown that the formation of various gold compounds during urea-assisted deposition–precipitation increases the loading capacity of gold nanoparticles [22,23]. However, Au agglomerates were formed during the calcination process when chloroauric acid was used to prepare supported nano-gold catalysts using the deposition–precipitation method due to residual chlorine. Washing the catalyst with ammonia can effectively prevent gold sintering by removing residual chlorine [16]. Therefore, a supported nano-gold catalyst was prepared using the deposition–precipitation method with urea (CO(NH_2_)_2_) (DPU) and washed with ammonia. The resulting supported nano-gold catalyst had a small particle size and uniform distribution.

Long-term SO_2_ exposure reduces the activity of supported nano-gold catalysts, severely limiting their application in treating pollutants in industrial flue gas [8]. The catalytic activity of supported nano-gold catalysts for CO oxidation significantly decreases in the presence of SO_2_ [24], possibly because of the increased adsorption strength between Au and CO after SO_2_ treatment, which inhibits the movement of adsorbed CO on Au particles to the gold–carrier interface to form CO_2_ [25]. The poor anti-sulphide ability of nano-gold catalysts is a crucial limiting factor in their application. Supported nano-Pd catalysts also exhibit strong CO catalytic oxidation performance [26,27], but they suffer from low activity at low temperatures [28]. Pd-Au bimetallic catalysts can be efficiently used in various catalytic reactions [29,30,31,32]. A theoretical calculation study reported by Jin Zhang et al. [33] showed that adding gold inhibits Pd/TiO_2_ poisoning in CO oxidation reactions by forming the “Golden Crown” Pd-Au structure. However, experimental studies investigating the effects of SO_2_ and H_2_S on the performance of supported Pd-Au catalysts have not been reported. Using in situ diffuse reflectance infrared Fourier transform spectroscopy (DRIFTS) and temperature programmed desorption (TPD), Wilburn et al. [34] showed that the adsorption of SO_2_ by Pd-Pt alloys depends on the Pd:Pt molar ratio and that the effect of SO_2_ and H_2_S on catalyst performance can be reduced by regulating the metal proportion in bimetallic catalysts.

The Al_2_O_3_ used in this study was spherical alumina. As an excellent carrier, it is the skeleton material of the Au/FeO_x_/Al_2_O_3_ catalyst, which is beneficial for the industrial application of catalysts and for preserving and filling in the catalyst reaction bed. Therefore, in this study, a Pd/FeO_x_/Al_2_O_3_ catalyst was prepared via ultrasonic-assisted impregnation using γ-Al_2_O_3_ as the first carrier and ferric oxide as the second carrier. Gold nanoparticles were prepared using a deposition–precipitation method. Pd-Au/FeO_x_/Al_2_O_3_ catalysts with a mass ratio of 1:2 (Pd:Au) were obtained. The loading capacity of Au nanoparticles was 2 wt%. The physicochemical properties of the Au/FeO_x_/Al_2_O_3_ and Pd-Au/FeO_x_/Al_2_O_3_ catalysts were compared using XRD, TEM, ICP, BET, XPS, and CO-TPR to evaluate catalytic CO oxidation activity and stability. The mechanism of catalytic CO oxidation and its adsorption on SO_2_ and H_2_S were calculated and analysed using density functional theory (DFT) studies and in situ FTIR, revealing the reasons for the more substantial anti-SO_2_ and anti-H_2_S effects of Pd-Au/FeO_x_/Al_2_O_3_.

## 2. Materials and Methods

### 2.1. Experimental Methods

#### 2.1.1. FeO_x_/Al_2_O_3_ Preparation

First, alumina was pre-treated with activation: γ-Al_2_O_3_ (size 1–3 mm) was placed in a crucible, heated in a muffle furnace at 600 °C and a rate of 10 °C/min, and kept at that temperature for 2 h. The FeO_x_/Al_2_O_3_ support was obtained by preparing a 0.4 mol/L solution of Fe(NO_3_)_3_ (AR) and supporting it on γ-Al_2_O_3_ using equal volume impregnation, followed by drying in an oven at 120 °C for 12 h. This support was then placed in a muffle furnace at 500 °C, with a heating rate of 10 °C/min, and maintained at this temperature for 2 h. This process was repeated for a 0.2 mol/L Fe(NO_3_)_3_ solution.

#### 2.1.2. Preparation of Au/FeO_x_/Al_2_O_3_ Catalysts

The FeO_x_/Al_2_O_3_ carrier was placed in a conical bottle with an equal weight of urea as the precipitator, followed by a solution of chloroauric acid with 2 wt% Au in 100 mL of deionised (DI) water. The resulting solution was stirred and heated to 80 °C. The heating was stopped when the pH reached 8–8.5. After 4 h, the catalyst precursor was washed with a large amount of deionised water and a small amount of ammonia water until no precipitation was observed when testing with a 0.5 mol/L silver nitrate solution. The washed catalyst was oven dried for 12 h at 120 °C. The dried samples were roasted in a tubular furnace in an oxygen atmosphere for 2 h at 300 °C and in a hydrogen atmosphere for 2 h at 300 °C to obtain a Au/FeO_x_/Al_2_O_3_ catalyst with 2 wt% Au. BET analysis of Au/FeO_x_/Al_2_O_3_ catalysts is shown in Figure S3a.

#### 2.1.3. Preparation of Pd-Au/FeO_x_/Al_2_O_3_ Catalysts

**Figure 1:**
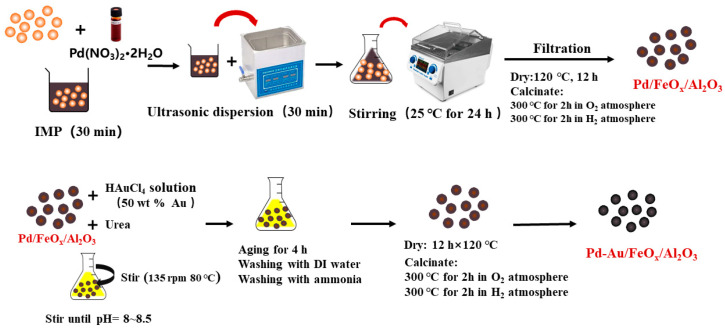
Preparation of Pd-Au/FeO_x_/Al_2_O_3_ catalysts.

After characterization analysis of Pd-Au/FeO_x_/A_2_O_3_ catalysts with 5 types of Pd: Au mass ratios (Table S1, Figures S1 and S2). Pd-Au/FeO_x_/Al_2_O_3_ catalysts were prepared, as shown in Figure 1.

(1) Preparation of Pd/FeO_x_/Al_2_O_3_ catalysts

Ten grams of FeO_x_/Al_2_O_3_ carriers was placed in a conical flask with 0.1 g of Pd(NO_3_)_2_ to obtain a final solution volume of 50 mL. After standing for 30 min and undergoing ultrasonic-assisted impregnation for 30 min, the solution was placed in a water bath shaker at 25 °C and 135 rpm for 24 h. After the reaction was completed, the product was dried for 12 h at 120 °C. The dried samples were roasted in a tube furnace at 300 °C in an oxygen atmosphere for 2 h and at 300 °C in a hydrogen atmosphere for 30 min to obtain Pd/FeO_x_/Al_2_O_3_ catalysts.

(2) Preparation of Pd-Au/FeO_x_/Al_2_O_3_ Catalysts

After step (1), The Pd/FeO_x_/Al_2_O_3_ catalysts were placed in a conical bottle with an equal weight of urea added as the precipitator, followed by a solution of chloroauric acid (2 wt% Au in 100 mL of deionised water). The solution was then stirred and heated to 80 °C. The heating was stopped when the pH reached 8–8.5. After 4 h, the obtained Pd-Au/FeO_x_/Al_2_O_3_ catalyst precursor was washed with a large amount of deionised water and a small amount of ammonia water until no precipitation was produced when testing with a 0.5 mol/L silver nitrate solution. The washed catalyst was oven dried for 12 h at 120 °C. The dried samples were roasted in a tubular furnace in an oxygen atmosphere for 2 h at 300 °C and in a hydrogen atmosphere for 2 h at 300 °C to obtain a Pd-Au/FeO_x_/Al_2_O_3_ catalyst with a Pd:Au load-mass ratio of 1:2. BET analysis of Pd-Au/FeO_x_/Al_2_O_3_ catalysts is shown in Figure S3b).

### 2.2. Test Methods

The catalyst structure was characterised using X-ray diffractometry (XRD, Panalytical, X’ Pert Pro MPD, Almelo, The Netherlands) at a scanning angle of 10–80° and a scanning rate of 2°/min, The wavelength was 1.5418 nm, the voltage was 40 kV, and the current was 40 mA. Transmission electron microscopy (TEM, JEM-F200, JEOL, Tokyo, Japan) at 200 kV was used to characterise the surface morphology and elemental distributions of the samples. An inductively coupled plasma optical emission spectrometer (ICP-OES, 5110, Agilent, Santa Clara, CA, USA) was used to determine the metal content of the catalysts. X-ray photoelectron spectroscopy (XPS, K-Alpha, Thermo Scientific, Waltham, MA, USA) was used to analyse the valence changes of the elements on the catalyst surface. The C1s binding energy was used to correct 284.8 eV. A temperature-programmed chemisorption instrument (CO-TPR, BELCat II, Microtrac, Osaka, Japan) was used to evaluate catalyst performance in CO oxidation; the samples were heated at 10 °C/min from room temperature to −300 °C for drying pretreatment. The air flow (50 mL/min) was purged for 1 h and then cooled to 50 °C. The samples were desorbed at 10 °C/min in 10% CO/He air flow and then desorbed at 900 °C, and the reduction gas was detected using TCD. In situ FTIR (80 V Brucker, Billerica, MA, USA) was used to detect and analyse the adsorption processes of the materials on the catalyst surface using the diffuse reflection integrating sphere model.

### 2.3. SO_2_ and H_2_S Pretreatment

For SO_2_ and H_2_S pretreatment, air was used as the balance gas before catalytic CO oxidation. The catalyst was treated with 2 ppm SO_2_ (100 mL/min for 1 h) and 2 ppm H_2_S (100 mL/min for 1 h) at 25 °C under atmospheric pressure. Air was used as the balance gas when evaluating the effect of temperature on the CO conversion rate; the CO concentration was 2500 ppm, and the flow rate was 50 mL/min. CO stability at room temperature (25 °C) was tested with a CO concentration of 20,000 ppm and a volume space velocity of 13,000 h^−1^.

### 2.4. Evaluation of Catalytic Activity

The catalytic oxidation of CO to CO_2_ was performed using a small fixed-bed continuous-flow reactor with mass flowmeter control of the raw gas flow and a U-shaped quartz tube with an inner diameter of 1 cm as the reaction tube. A supported nano-gold catalyst (0.5 g) was weighed into the reaction tube, and the catalyst bed was fixed using quartz cotton at both ends. The raw gas consisted of 0.25% CO, 21.1% N_2_, and 78.5% O_2_. The flow rate was 50 mL/min, and the reaction temperature was −30–60 °C. Gas chromatography using an HP6890 gas chromatograph with high-purity hydrogen as the carrier gas, a hydrogen ion flame detector (FID), and a thermal conductivity detector (TCD) containing a reformer was used to detect the concentration of CO in the tail gas. The CO conversion rate (X_CO_, %) was calculated using the following equation:

(1) $$X_{CO}(\%)=\left(1-\frac{C_{out}}{C_{in}}\right)\times 100\%,$$

where C_in_ and C_out_ represent the initial CO concentration and the CO concentration in the tail gas_,_ respectively.

### 2.5. Evaluation of the Stability of the Catalysts

**Figure 2:**
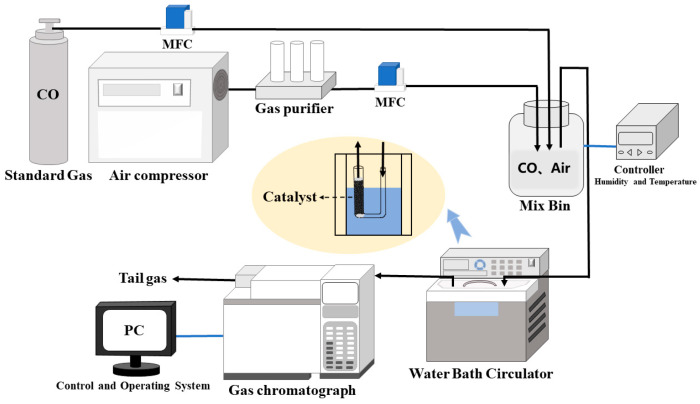
Schematic for evaluating catalyst stability.

Catalyst stability was also evaluated using a small fixed-bed continuous-flow reaction device, as shown in Figure 2. Here, the CO standard gas was diluted with a specific flow of compressed air to control the rate of CO generation. A U-shaped hard quartz tube with an inner diameter of 1 cm was used as the reaction tube. The height of the catalyst bed in the reaction tube was 8 cm, the bed density was 0.64 g/cm^3^, the volume space velocity was 13,000 h^−1^, the reaction temperature was 25 °C, and the concentration was 20,000 ppm. The concentrations of CO and CO_2_ in the tail gas were determined using gas chromatography (HP6890).

### 2.6. DFT Calculations

All DFT calculations were performed using the Vienna ab initio simulation (VASP5.4.4) code [35]. The exchange correlation was simulated with the PBE functional, and the ion–electron interactions were described using the projector augmented wave (PAW) method [36,37] Van der Waals (vdW) interactions were included using the empirical DFT-D3 method [38]. The Fe_2_O_3_ (110) surface supported the Au_13_ and Au_9_Pd_4_ nanoclusters. The atoms in the upper two layers of the surface were allowed to move freely, whereas the bottom two layers were fixed to simulate the surface of the structure. The Monkhorst–Pack-grid-mesh-based Brillouin zone k-points were set to 2 × 2 × 1 for all periodic structures, with a cut-off energy of 450 eV. The convergence criteria were 0.01 eV A^−1^ and 10^−5^ eV in force and energy, respectively. 

The free energy for species adsorption (ΔG) was calculated using the following equation:

(2) $$\Delta G=\Delta E+\Delta E_{\mathrm{ZPE}}+\Delta H_{0\to T}-T\Delta S,$$

where ΔE, ΔE_ZPE_, and ΔS represent the changes in electronic energy, zero-point energy, and entropy, respectively, caused by intermediate adsorption. ΔH_0→T_ represents the change in enthalpy when heated from 0 to T K.

## 3. Results and Discussion

### 3.1. Physical and Chemical Characterization of Catalysts

#### 3.1.1. XRD Analysis

**Figure 3:**
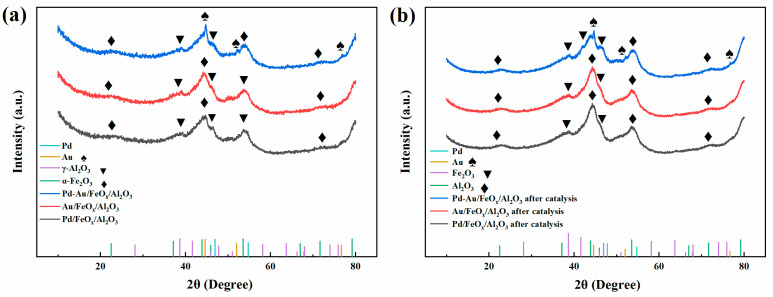
XRD patterns of Pd/FeO_x_/Al_2_O_3_, Au/FeO_x_/Al_2_O_3_, and Pd-Au/FeO_x_/Al_2_O_3_ catalysts. Before catalysis (**a**) and after catalysis (**b**).

Figure 3 shows the XRD patterns of Pd/FeO_x_/Al_2_O_3_, Au/FeO_x_/Al_2_O_3_ and Pd-Au/FeO_x_/Al_2_O_3_ catalysts. The Au/FeO_x_/Al_2_O_3_ and Pd-Au/FeO_x_/Al_2_O_3_ catalysts exhibited characteristic peaks of γ-Al_2_O_3_ and α-Fe_2_O_3_ [39,40]. However, AuNPs were not detected because they were highly dispersed on the FeO_x_/Al_2_O_3_ support. In the Pd-Au/FeO_x_/Al_2_O_3_ catalyst, both the characteristic peaks of γ-Al_2_O_3_ and α-Fe_2_O_3_ and the characteristic peaks of gold nanoparticles (44.6°, 52.0°, and 76.7° [41]) were detected, possibly because of the effect of Pd on the dispersion of gold nanoparticles. As the XRD pattern of this bimetallic catalyst did not show the characteristic peak of Pd, we confirmed its loading onto the FeO_x_/Al_2_O_3_ carrier through HRTEM characterisation and mapping of the energy spectrum. Moreover, after catalysis, the structures of these three catalysts remained unchanged.

#### 3.1.2. HRTEM Analysis

**Figure 4:**
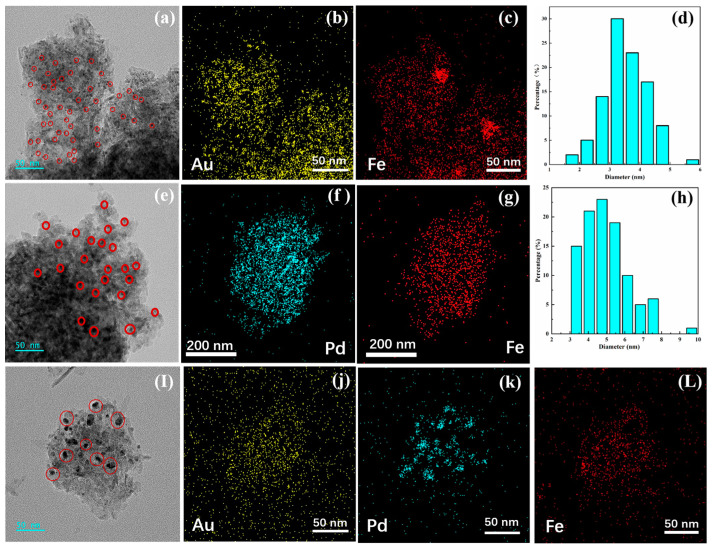
HRTEM diagram of the Au/FeO_x_/Al_2_O_3_ catalyst (**a**), with characterisation of its Au and Fe elemental distribution (**b**,**c**) and Au particle size distribution (**d**). HRTEM diagram of the Pd/FeO_x_/Al_2_O_3_ catalyst (**e**), with characterisation of its Pd and Fe elemental distribution (**f**,**g**) and Pd particle size distribution (**h**). HRTEM diagram of the Pd-Au/FeO_x_/Al_2_O_3_ catalyst (**I**), with characterisation of Au, Pd, and Fe elemental distribution (**j**–**L**).

**Figure 5:**
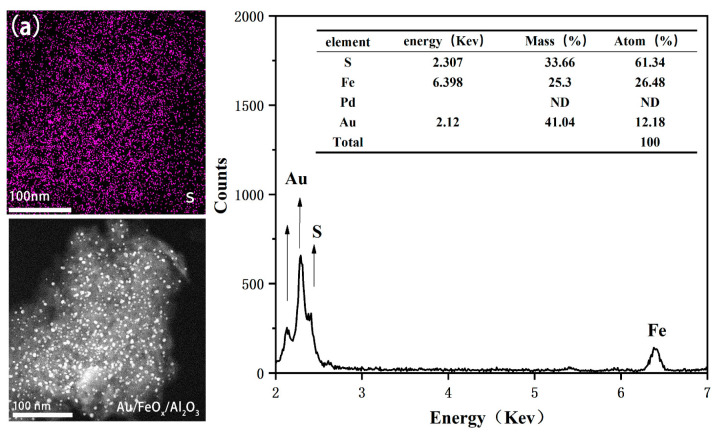

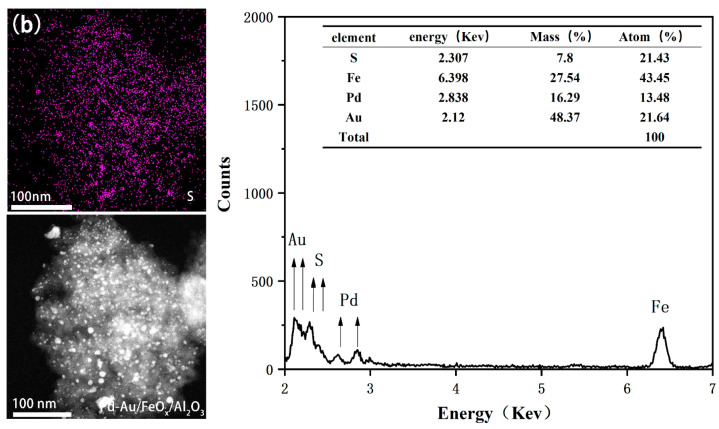
XEDS spectra of Au/FeO_x_/Al_2_O_3_ (**a**) and Pd-Au/FeO_x_/Al_2_O_3_ (**b**). The upper left corner of each figure is the mapping diagram of S.

Figure 4 shows HRTEM images and elemental distribution diagrams of the prepared Au/FeO_x_/Al_2_O_3_, Pd/FeO_x_/Al_2_O_3_, and Pd-Au/FeO_x_/Al_2_O_3_ catalysts. Au was uniformly dispersed in Au/FeO_x_/Al_2_O_3_ (Figure 4a,b). The particle size distribution of the Au nanoparticles was measured using Nano Measurer software, revealing an average particle size of 3.5 nm (Figure 4d). Pd nanoparticles were uniformly dispersed in Au/FeO_x_/Al_2_O_3_ (Figure 4e,f). Pd nanoparticles in the Pd-Au/FeO_x_/Al_2_O_3_ catalyst had a larger particle size (Figure 4I,k), with smaller nanometre Au particles on the Pd particles. XEDS atlas and S element mapping were performed for the sulphurated Au/FeO_x_/Al_2_O_3_ and Pd-Au/FeO_x_/Al_2_O_3_ catalysts under similar conditions (Figure 5), revealing an S adsorption of 7.8 and 33.66 wt% onto Pd-Au/FeO_x_/Al_2_O_3_ and Au/FeO_x_/Al_2_O_3_ catalysts, respectively. This confirms that SO_2_ and H_2_S are easily adsorbed onto the Au/FeO_x_/Al_2_O_3_ catalyst. HRTEM of Au/FeO_x_/Al_2_O_3_ and Pd-Au/FeO_x_/Al_2_O_3_ after reaction with CO are shown in Figures S4 and S5.

#### 3.1.3. XPS Analysis 

**Figure 6:**
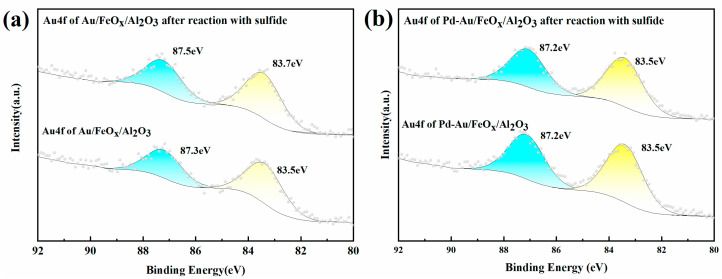
Au4f XPS of Au/FeO_x_/Al_2_O_3_ (**a**) and Pd-Au/FeO_x_/Al_2_O_3_ (**b**) before and after SO_2_ and H_2_S treatment.

**Figure 7:**
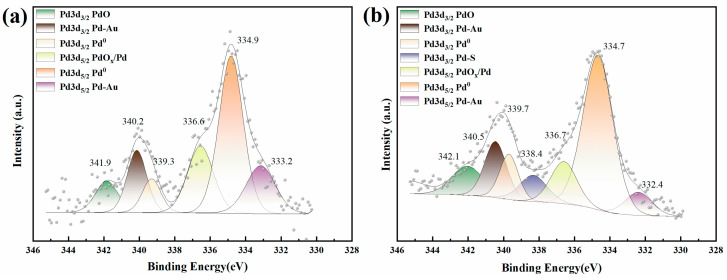
XPS spectra of Pd-Au/FeO_x_/Al_2_O_3_ before (**a**) and after SO_2_ and H_2_Streatment (**b**).

XPS spectra were used to analyse the Au4f and Pd3d valence states of Au/FeO_x_/Al_2_O_3_ and Pd-Au/FeO_x_/Al_2_O_3_ after treatment with SO_2_ and H_2_S (Figure 6 and Figure 7). Figure 6 shows the Au4f binding spectra of both catalysts. The peaks of Au/FeO_x_/Al_2_O_3_ at 87.3 eV and 83.5 eV were assigned to Au4f 5/2 and Au4f 7/2, respectively, both of which corresponded to Au^0^ (Figure 6a) [42,43]. After vulcanisation, the peaks corresponding to Au4f 5/2 and Au4f 7/2 increased in energy, appearing at 87.5 eV and 83.7 eV, respectively. This may be caused by the reaction of SO_2_ and H_2_S with gold nanoparticles to form Au^δ+^. For Pd-Au/FeO_x_/Al_2_O_3_, the Au4f 5/2 and Au4f 7/2 peaks remained unchanged with SO_2_ and H_2_S addition (Figure 6b), indicating that the addition of Pd weakened the effect of SO_2_ and H_2_S on the Au nanoparticles.

Figure 7 shows the Pd3d binding energy spectrum of the Pd-Au/FeO_x_/Al_2_O_3_ catalyst. Without SO_2_ and H_2_S treatment, the Pd3d 3/2 peaks appeared in the 339–342 eV range, with peaks at 341.9, 340.2, and 339.3 eVs corresponded to PdO, Pd, and Pd^0^ of the interacting Pd-Au species [43,44,45]. The Pd3d 5/2 peaks appeared in the 333–337 eV range, with peaks at 336.6, 334.9, and 333.2 eV attributed to PdO_x_/Pd, Pd^0^, and Pd of the interacting Pd-Au species, respectively [45,46,47]. The Pd3d 3/2 peaks of Pd-Au/FeO_x_/Al_2_O_3_ after SO_2_ and H_2_S treatment appeared at 338–342 eV. The peaks at 342.1, 340.5, 339.7, and 338.4 eV corresponded to PdO, Pd, and Pd^0^ in Pd-Au interacting with SO_2_ and H_2_S, and Pd in Pd-S interacting with SO_2_ and H_2_S [46]. The peaks in the 332–347 eV range were attributed to Pd3d 5/2, with peaks at 336.7, 334.7, and 332.4 eV, corresponding to PdO_x_/Pd, Pd^0^, and Pd of the interacting Pd-Au species [44,47]. The binding energy of Pd-S interacting with SO_2_ and H_2_S increased after SO_2_ and H_2_S treatment because the SO_2_ and H_2_S adsorbed to Pd to form Pd-S [48]. By calculating the content of Pd in different states before and after SO_2_ and H_2_S treatment (Table 1), we determined that Pd-Au and PdO_x_/Pd decreased after SO_2_ and H_2_S treatment, whereas the Pd^0^ content increased. We hypothesised that this was due to PdO_x_ reduction to Pd^0^ in the presence of SO_2_ and H_2_S. The Pd in Pd-Au exhibited stronger adsorption with SO_2_ and H_2_S [49,50].

### 3.2. CO Oxidation Catalyst Performance

#### 3.2.1. Evaluating Catalytic Activity

**Figure 8:**
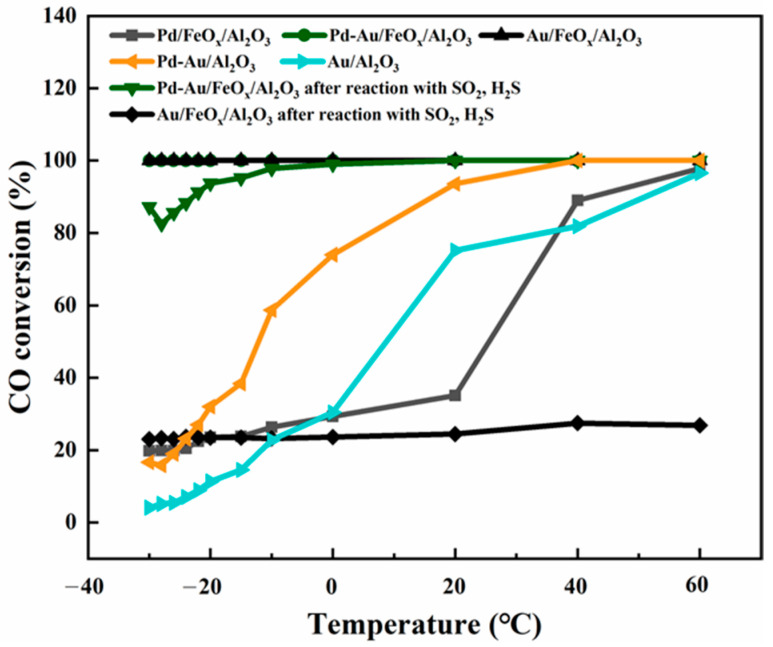
CO conversion rates at varying temperatures by Pd/FeO_x_/Al_2_O_3_, Pd-Au/Al_2_O_3_, and Au/Al_2_O_3_. CO conversion rates at varying temperatures by Au/FeO_x_/Al_2_O_3_ and Pd-Au/FeO_x_/Al_2_O_3_ catalysts before and after SO_2_ and H_2_S pretreatment.

Figure 8 shows the CO conversion for the catalytic oxidation of CO at different temperatures using Au/FeO_x_/Al_2_O_3_ and Pd-Au/FeO_x_/Al_2_O_3_ as catalysts before and after SO_2_ and H_2_S pretreatment. Compared with Pd/FeO_x_/Al_2_O_3_ catalysts, the activities of Pd-Au/FeO_x_/Al_2_O_3_ and Au/FeO_x_/Al_2_O_3_ catalysts were significantly improved. Compared with Au/Al_2_O_3_ and Pd-Au/Al_2_O_3_ catalysts, after the addition of FeO_x_, the activities of Pd-Au/FeO_x_/Al_2_O_3_ and Au/FeO_x_/Al_2_O_3_ catalysts were significantly improved. The Pd-Au/FeO_x_/Al_2_O_3_ and Au/FeO_x_/Al_2_O_3_ catalysts without SO_2_ and H_2_S pretreatment had excellent CO conversion rates, achieving complete conversion at a CO concentration of 2500 ppm in the −30–60 °C temperature range. After SO_2_ and H_2_S pretreatment, the Pd-Au/FeO_x_/Al_2_O_3_ catalyst conversion rate for a CO concentration of 2500 ppm was 87.2% at −30 °C, and the conversion rate was 100% for temperatures above 25 °C. However, the conversion rate of Au/FeO_x_/Al_2_O_3_ after SO_2_ and H_2_S pretreatment was only 23–26% at all tested temperatures, indicating that the Pd-Au/FeO_x_/Al_2_O_3_ catalyst was affected less by SO_2_ and H_2_S, matching the observed low SO_2_ and H_2_S adsorption measured by XEDS.

#### 3.2.2. Evaluating Catalyst Stability 

**Figure 9:**
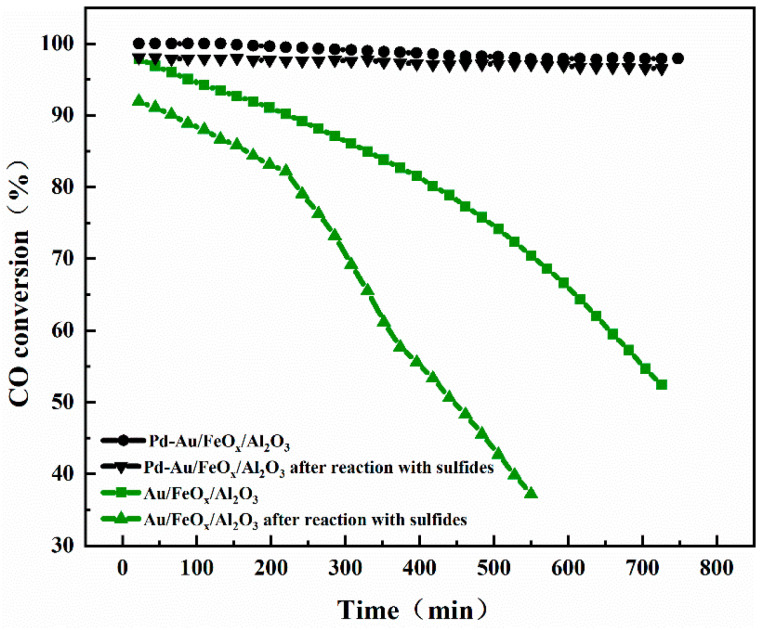
Stability test of CO oxidation by Au/FeO_x_/Al_2_O_3_ and Pd-Au/FeO_x_/Al_2_O_3_ catalysts before and after SO_2_ and H_2_S pretreatment.

Figure 9 shows the catalytic oxidation stability test curves of the Au/FeO_x_/Al_2_O_3_ and Pd-Au/FeO_x_/Al_2_O_3_ catalysts for CO at 25 °C before and after SO_2_ and H_2_S treatment. The conversion rate of the Au/FeO_x_/Al_2_O_3_ catalyst decreased from 97.9% to 52.4% after 726 min, which was slower than that of the Au/FeO_x_/Al_2_O_3_ catalyst pretreated with SO_2_ and H_2_S (91.9% to 37% after 550 min). The conversion rate of Pd-Au/FeO_x_/Al_2_O_3_ was 100% in the first 132 min, the TON of it was 205, and it declined slowly to 97.9% after 748 min. Similarly, the conversion rate of SO_2_- and H_2_S-pretreated catalyst decreased from 98.1% to 96.5% after 726 min. Thus, Pd-Au/FeO_x_/Al_2_O_3_ maintained strong stability after SO_2_ and H_2_S treatment, with negligible conversion reduction (approximately 2%) after 726 min. It is deduced that this is due to the adsorption of SO_2_ and H_2_S on the catalyst surface, which inhibits the adsorption and reaction of CO.

#### 3.2.3. Mechanistic Analysis of CO Oxidation

**Figure 10:**
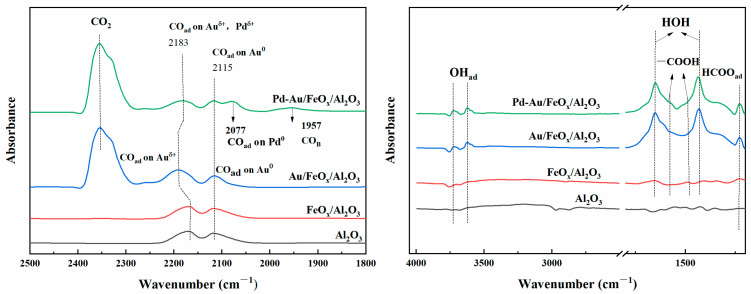
In situ FTIR spectra of Au/FeO_x_/Al_2_O_3_ and Pd-Au/FeO_x_/Al_2_O_3_ catalysts with 2 wt% CO at 25 °C.

Figure 10 shows the in situ FTIR spectra of Al_2_O_3_, FeO_x_/Al_2_O_3_, Au/FeO_x_/Al_2_O_3_, and Pd-Au/FeO_x_/Al_2_O_3_ with a 2% CO mass fraction. Both the Pd-Au/FeO_x_/Al_2_O_3_ and Au/FeO_x_/Al_2_O_3_ catalysts exhibited peaks corresponding to -OH (3600–3700 cm^−1^), -COOH (1300–1700 cm^−1^), CO (~1900 cm^−1^, 2000–2200 cm^−1^), and CO_2_ (2300–2400 cm^−1^) vibrations [51,52]. -OH represents water adsorbed on the catalyst surface and reacting with the catalyst surface active oxygen species. CO_ad_ reacted with -OH_ad_ to produce -COOH, which adsorbed on the catalyst surface [53]. The peak intensities of -OH and -COOH on Pd-Au/FeO_x_/Al_2_O_3_ were similar to those of Au/FeO_x_/Al_2_O_3_. However, for the Pd-Au/FeO_x_/Al_2_O_3_ catalyst, CO was adsorbed on Pd^0^, Pd^δ+^, Au^0^, and Au^δ+^. The peak at 2077 cm^−1^ corresponded to CO adsorption on Pd^0^ [52]. The peak of adsorbed CO on the Pd-Au/FeO_x_/Al_2_O_3_ catalyst (2183 cm^−1^) had a higher frequency than that of Au^δ+^ on the Au/FeO_x_/Al_2_O_3_ catalyst (2190 cm^−1^), possibly because of overlapping peaks [51,52,54,55,56]. Bridge adsorption (CO_B_) of CO occurs at 1957 cm^−1^; that is, CO is adsorbed on the metal centre of Au and Pd [53,57]. Based on the literature and in situ FTIR measurements, we concluded the reaction path of CO adsorbed at the active centre of the catalyst (Au^0^, Au^δ+^, Pd^0^, and Pd^δ+^), reacting with adsorbed water to produce CO_2_ and -COOH, while -COOH partially accumulated on the catalyst’s surface and partially reacted with oxygen on the catalyst’s surface to produce CO_2_ and release gradually [53].

**Figure 11:**
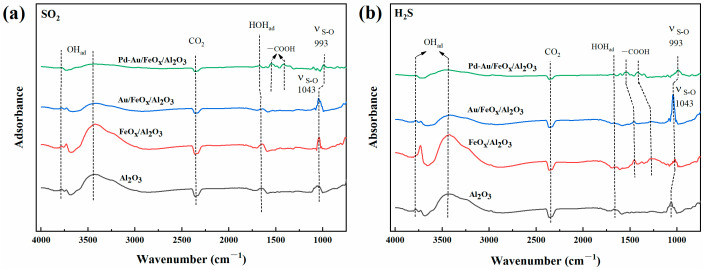
In situ FTIR spectra of the support, Au/FeO_x_/Al_2_O_3_, and Pd-Au/FeO_x_/Al_2_O_3_ treated with SO_2_ (**a**) and H_2_S (**b**) at 25 °C.

Figure 11 shows an in situ FTIR diagram of Au/FeO_x_/Al_2_O_3_ and Pd-Au/FeO_x_/Al_2_O_3_ after treatment with 2 ppm SO_2_ for 1 h and 2 ppm H_2_S for 1 h at ambient temperature (25 °C). The surfaces of the carrier and catalyst formed -OH (3400–3800 cm^−1^), -COOH (1400–1600 cm^−1^), H_2_O (1600 cm^−1^), and S-O (~1000 cm^−1^) bonds [58,59,60]. Since there was the presence of air in the reaction gas, the negative peak in the figure was CO_2_ in the air. During SO_2_ adsorption, S-O bonds were observed on the surface of both the carrier and catalyst, classified as SO_4_^2−^ [58]. The SO_4_^2−^ peak of Pd-Au/FeO_x_/Al_2_O_3_ was smaller than that of Au/FeO_x_/Al_2_O_3_, indicating a more difficult SO_2_ adsorption to form SO_4_^2−^ groups on the surface. After H_2_S treatment, the intensity of the peak corresponding to S-O vibration in Au/FeO_x_/Al_2_O_3_ increased significantly. In contrast, there was no significant variation in Pd-Au/FeO_x_/Al_2_O_3_, indicating that this catalyst had improved anti-SO_2_ and anti-H_2_S adsorption ability. However, the effect of H_2_S on the catalyst was not clear because the test method in this paper cannot be analysed for the time being. After sulphide treatment, the surface of the catalyst and carrier adsorbed -COOH, which was generated by the reaction of the CO component in the mixture with adsorbed water [53,57].

**Figure 12:**
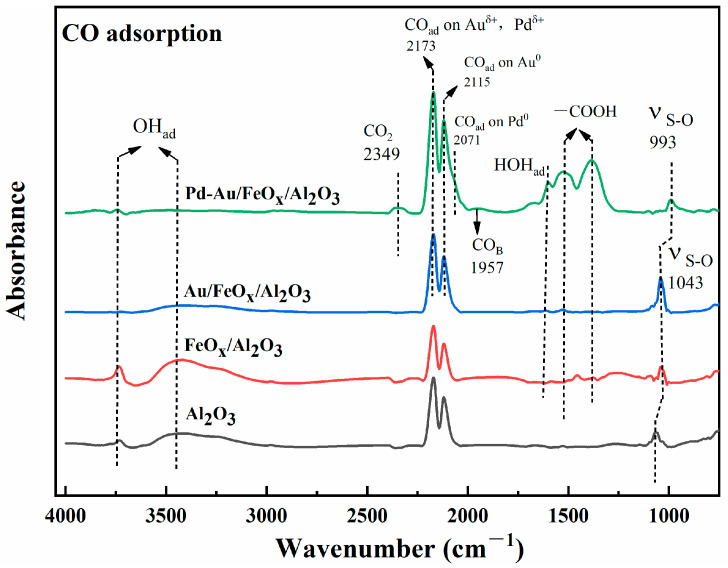
In situ FTIR spectra of CO adsorption on Au/FeO_x_/Al_2_O_3_ and Pd-Au/FeO_x_/Al_2_O_3_ catalysts after SO_2_ and H_2_S pretreatment at 25 °C.

After SO_2_ and H_2_S pretreatment, air was used as the equilibration gas. A mixture with 20,000 ppm of CO was injected, and the material was characterised by in situ FTIR (Figure 12). The vibration peak of CO increased after CO was injected into the support, Au/FeO_x_/Al_2_O_3_, and Pd-Au/FeO_x_/Al_2_O_3_. In contrast, the vibration peak of CO_2_ (2349 cm^−1^) only increased for the Pd-Au/FeO_x_/Al_2_O_3_ catalyst. On the Pd-Au/FeO_x_/Al_2_O_3_ catalyst, bridging CO was adsorbed on the active sites of Au and Pd at 1956 cm^−1^ [53], and the vibration peak at 2071 cm^−1^ corresponded to CO adsorbed on Pd^0^. The vibration peak at 2115 cm^−1^ corresponded to CO adsorbed on Au^0^. The vibration peak at 2173 cm^−1^ corresponded to Au^δ+^ and Pd^δ+^ [52,57]. Thus, a large amount of CO was adsorbed on the bimetallic active sites of the Pd-Au/FeO_x_/Al_2_O_3_ catalyst, reacting to produce CO_2_. In contrast, no CO_2_ production was observed on the Au/FeO_x_/Al_2_O_3_ catalyst, possibly because the sulphate species adsorbed and accumulated on the Au/FeO_x_/Al_2_O_3_ catalyst inhibited the reaction of CO with the active centre of the catalyst. Additionally, the peak corresponding to the vibration of -COOH adsorbed on Pd-Au/FeO_x_/Al_2_O_3_ was derived from the reaction of CO adsorbed on the catalyst surface with reactive oxygen species and absorbed water [53].

#### 3.2.4. DFT Calculations

**Figure 13:**
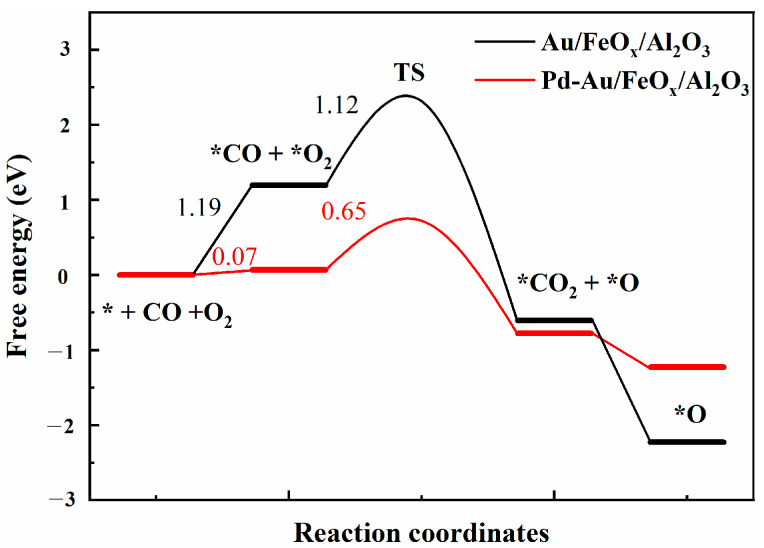
Free energy curves of CO oxidation catalysed by Au/FeO_x_/Al_2_O_3_ and Pd-Au/FeO_x_/Al_2_O_3_.

Maumau T.R. et al. [61] compared the electro-oxidation activity of Pd/C, Au/C, Pd (Au/C), and Pd-Au/C catalysts to alcohols and found that Pd-Au/C catalysts were more stable and more toxic tolerant. Comparing the reaction kinetics of Pd-Au/SiO_2_ and Pd/SiO_2_ catalysts, Han Y.F. et al. [62] showed that the decrease in reactant adsorption on the catalyst and the enhancement of surface adsorbed oxygen/adsorbed oxygen mobility were the main reasons for the enhancement of Pd-Au/SiO_2_ catalyst activity. Gao feng et al. [63] compared Pd-Au particles supported on different carriers and applied reaction kinetics analysis to find that the Pd-Au-supported catalyst was more likely to oxidize and lose activity than the Pd-Au-supported catalyst, and Pd-Au was more likely to desorb CO, which was also the main reason for the enhanced activity of the Pd-Au-supported catalyst. As shown in Figure 13, the reaction free energy curve calculated using DFT shows that *CO and *O_2_ adsorption on Au/FeO_x_/Al_2_O_3_ was very weak, with an energy change of 1.19 eV. Pd-Au/FeO_x_/Al_2_O_3_ had a lower free energy of 0.07 eV. Regarding the transition state, the energy barriers corresponding to *CO to *CO_2_ oxidation by Au/FeO_x_/Al_2_O_3_ and Pd-Au/FeO_x_/Al_2_O_3_ catalysts were 1.12 eV and 0.65 eV, respectively, indicating that Pd-Au/FeO_x_/Al_2_O_3_ is thermodynamically more likely to oxidise CO.

**Figure 14:**
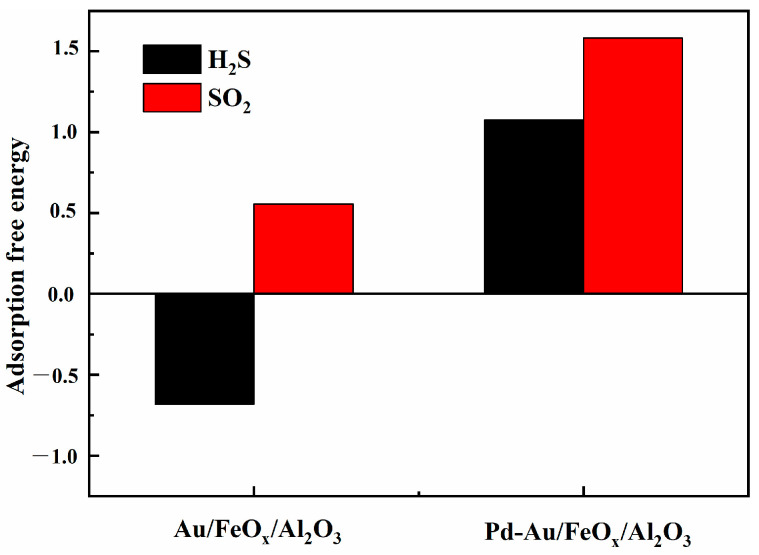
Adsorption energies of SO_2_ and H_2_S on Au/FeO_x_/Al_2_O_3_ and Pd-Au/FeO_x_/Al_2_O_3_ catalysts.

Finally, the antivulcanisation of Au/FeO_x_/Al_2_O_3_ and Pd-Au/FeO_x_/Al_2_O_3_ was investigated. The adsorption energy diagrams of H_2_S and SO_2_ were obtained from the difference in SO_2_ and H_2_S adsorption energies (Figure 14). Higher adsorption energy corresponded to more difficult SO_2_ and H_2_S adsorption. The adsorption energy of Pd-Au/FeO_x_/Al_2_O_3_ was positive for both H_2_S and SO_2_, whereas that of Au/FeO_x_/Al_2_O_3_ for H_2_S was negative. The adsorption energy of Au/FeO_x_/Al_2_O_3_ for SO_2_ was also significantly lower than that of Pd-Au/FeO_x_/Al_2_O_3_. Thus, Pd-Au/FeO_x_/Al_2_O_3_ has a weak adsorption capacity for SO_2_ and H_2_S and a more substantial anti-SO_2_ and anti-H_2_S effect, indicative of its long-term stability in a sulphur-containing environment.

## 4. Conclusions

In this study, a Pd-Au/FeO_x_/Al_2_O_3_ catalyst with an Au:Pd ratio of 2:1 (wt%) was prepared using ultrasonic-assisted impregnation and urea-assisted deposition–precipitation methods, and its performance was compared with that of Au/FeO_x_/Al_2_O_3_. XRD and TEM analyses showed that the prepared Au and Pd nanoparticles were evenly dispersed on the carrier. Mapping and XEDS analyses showed that the Pd-Au/FeO_x_/Al_2_O_3_ catalyst adsorbed less SO_2_ and H_2_S after SO_2_ and H_2_S pretreatment and had stronger SO_2_ and H_2_S resistance than Au/FeO_x_/Al_2_O_3_. The Pd-Au/FeO_x_/Al_2_O_3_-catalysed CO conversion was measured before and after SO_2_ and H_2_S pretreatment, revealing that the Pd-Au/FeO_x_/Al_2_O_3_ catalyst could fully convert 2500 ppm of CO at 25 °C (room temperature) after SO_2_ and H_2_S pretreatment. The conversion rate remained at 100% at higher temperatures. Stability tests of the two catalysts before and after SO_2_ and H_2_S pretreatment showed that the conversion rate of Pd-Au/FeO_x_/Al_2_O_3_ with 20,000 ppm of CO was 98.1% at 25 °C (ambient temperature) after SO_2_ and H_2_S pretreatment, with a less than 2% decrease after 726 min. In situ FTIR analysis of the Pd-Au/FeO_x_/Al_2_O_3_ catalyst showed that CO can be adsorbed on Pd and Au as well as bridging the two metals. The roles of the two catalysts in catalytic CO oxidation were studied using DFT calculations. The results showed that the conversion to CO_2_ on Pd-Au/FeO_x_/Al_2_O_3_ required less energetic CO and O_2_ species. By comparing the adsorption energies of SO_2_ and H_2_S on the two catalysts, we concluded that the Pd-Au/FeO_x_/Al_2_O_3_ catalyst was less susceptible to the activity-reducing SO_2_ and H_2_S effect. Thus, the addition of Pd to Au/FeO_x_/Al_2_O_3_ catalysts resulted in more robust catalytic CO oxidation activity and improved anti-SO_2_ and anti-H_2_S stability. Pd-Au/FeO_x_/Al_2_O_3_ has the potential for wide use in the treatment of industrial flue gases.

## Figures and Tables

**Table 1:** Pd3d contents in different valence states before and after SO_2_ and H_2_S treatment.

	Pd 3d _3/2_			Pd 3d _5/2_			
	PdO	Pd-Au	Pd^0^	Pd-S	PdO_x_/Pd	Pd^0^	Pd-Au
Pd-Au/FeO_x_/Al_2_O_3_ (Atom, %)	9.84	14.95	13.76	\	14.81	20.36	26.29
Pd-Au/FeO_x_/Al_2_O_3_ after reacting with sulphides (Atom, %)	9.02	12.01	8.26	7.07	11.18	46.11	6.36

## Data Availability

Not applicable.

## Supplementary Materials

The following supporting information can be downloaded at: https://www.mdpi.com/article/10.3390/ma16103755/s1, Table S1: Au and Pd mass loading of catalysts; Figure S1: CO conversion of Pd-Au/FeO_x_/Al_2_O_3_ catalysts with different mass loading ratios; Figure S2: CO conversion of Pd-Au/FeO_x_/Al_2_O_3_ catalysts with different mass loading ratios after sulphide pretreatment; N_2_ adsorption-desorption isotherms and pore size distributions of Au/FeO_x_/Al_2_O_3_ (a) and Pd-Au/FeO_x_/Al_2_O_3_ (b); Figure S4 HRTEM of Au/FeO_x_/Al_2_O_3_ catalyst; Figure S5 HRTEM of Pd- Au/FeO_x_/Al_2_O_3_ catalyst.

## References

[1] Rose J.J., Wang L., Xu Q.Z., McTiernan C.F., Shiva S., Tejero J., Gladwin M.T. (2017). Carbon Monoxide Poisoning: Pathogenesis, Management, and Future Directions of Therapy. Am. J. Respir. Crit. Care Med..

[2] Zhang Q., Guan J. (2022). Applications of single-atom catalysts. Microchem. J..

[3] Xie X., Zhang X., Xie M., Xiong L., Sun H., Lu Y., Mu Q., Rummeli M.H., Xu J., Li S. (2022). Au-activated N motifs in non-coherent cupric porphyrin metal organic frameworks for promoting and stabilizing ethylene production. Nat. Commun..

[4] Qin L., Wang Z., Fu Y., Lai C., Liu X., Li B., Liu S., Yi H., Li L., Zhang M. (2021). Gold nanoparticles-modified MnFe_2_O_4_ with synergistic catalysis for photo-Fenton degradation of tetracycline under neutral pH. J. Hazard. Mater..

[5] Liu H., Cheng J., He W., Li Y., Mao J., Zheng X., Chen C., Cui C., Hao Q. (2022). Interfacial electronic modulation of Ni_3_S_2_ nanosheet arrays decorated with Au nanoparticles boosts overall water splitting. Appl. Catal. B Environ..

[6] Jiang X., Huang J., Bi Z., Ni W., Gurzadyan G., Zhu Y., Zhang Z. (2022). Plasmonic Active “Hot Spots”-Confined Photocatalytic CO_2_ Reduction with High Selectivity for CH_4_ Production. Adv. Mater..

[7] Jiang W., Low J., Mao K., Duan D., Chen S., Liu W., Pao C.-W., Ma J., Sang S., Shu C. (2021). Pd-Modified ZnO-Au Enabling Alkoxy Intermediates Formation and Dehydrogenation for Photocatalytic Conversion of Methane to Ethylene. J. Am. Chem. Soc..

[8] Feng C., Liu X., Zhu T., Tian M. (2021). Catalytic oxidation of CO on noble metal-based catalysts. Environ. Sci. Pollut. Res..

[9] Wu F., He L., Li W.C., Lu R., Wang Y., Lu A.H. (2021). Highly dispersed boron-nitride/CuO_x_-supported Au nanoparticles for catalytic CO oxidation at low temperatures. Chin. J. Catal..

[10] Amrute A.P., De Bellis J., Felderhoff M., Schuth F. (2021). Mechanochemical Synthesis of Catalytic Materials. Chem.-A Eur. J..

[11] Zhang Z., Tang Y., Du W., Xu J., Wang Q., Song N., Qian G., Duan X., Zhou X. (2022). Engineering gold impregnated uncalcined TS-1 to boost catalytic formation of propylene oxide. Appl. Catal. B Environ..

[12] Nishio H., Miura H., Kamata K., Shishido T. (2021). Deposition of highly dispersed gold nanoparticles onto metal phosphates by deposition-precipitation with aqueous ammonia. Catal. Sci. Technol..

[13] Zhu D., Xu Y., Shi J., Zou X., Zhang W., Huang X., Li Z. (2021). Selective enrichment and electrochemical determination of Cu in mushroom using L-Cysteine functionalized Fe_3_O_4_@Au nanoparticles. Microchem. J..

[14] Nakahara R., Sakai M., Kimura T., Yamamoto M., Syouji A., Hara K., Kouno T. (2021). Lasing action of ZnO nanowires grown by mist chemical vapor deposition using thin Au layer on c-plane sapphire substrate. Jpn. J. Appl. Phys..

[15] Li Z., Wu L., Guo J., Shao Y., Song Y., Ding Y., Zhu L., Yao X. (2021). Light-Promoted Minisci Coupling Reaction of Ethers and Aza Aromatics Catalyzed by Au/TiO_2_ Heterogeneous Photocatalyst. ChemCatChem.

[16] Dobrosz-Gomez I., Gomez-Garcia M.A., Rynkowski J.M. (2020). The Origin of Au/Ce_1-x_Zr_x_O_2_ Catalyst’s Active Sites in Low-Temperature CO Oxidation. Catalysts.

[17] Yang S.S., Huang Z.Y., Wu P.X., Li Y.H., Dong X.B., Li C.Q., Zhu N.Y., Duan X.D., Dionysiou D.D. (2020). Rapid removal of tetrabromobisphenol A by alpha-Fe_2_O_3-x_@Graphene@Montmorillonite catalyst with oxygen vacancies through peroxymonosulfate activation: Role of halogen and alpha-hydroxyalkyl radicals. Appl. Catal. B Environ..

[18] Zhang S.C., Liu Z.F., Chen D., Yan W.G. (2020). An efficient hole transfer pathway on hematite integrated by ultrathin Al_2_O_3_ interlayer and novel CuCoO_x_ cocatalyst for efficient photoelectrochemical water oxidation. Appl. Catal. B Environ..

[19] Yang J., Hu S.Y., Fang Y.R., Hoang S., Li L., Yang W.W., Liang Z.F., Wu J., Hu J.P., Xiao W. (2019). Oxygen Vacancy Promoted O_2_ Activation over Perovskite Oxide for Low-Temperature CO Oxidation. ACS Catal..

[20] Ma G.Y., Wang L., Wang X.R., Li L., Ma H.F. (2022). CO Oxidation over Alumina-Supported Copper Catalysts. Catalysts.

[21] Wang C.L., Yao Q., Cao L.N., Li J.J., Chen S., Lu J.L. (2019). Precise Tailoring of Ir-FeOx Interfaces for Improved Catalytic Performance in Preferential Oxidation of Carbon Monoxide in Hydrogen. J. Phys. Chem. C.

[22] Rio E.D., Blanco G., Collins S., Haro M.L., Chen X.W., Delgado J.J., Calvino J.J., Bernal S. (2011). CO Oxidation Activity of a Au/Ceria-Zirconia Catalyst Prepared by Deposition-Precipitation with Urea. Top. Catal..

[23] Chrouda A., Ahmed S.M.A., Elamin M.B. (2022). Preparation of Nanocatalysts Using Deposition Precipitation with Urea: Mechanism, Advantages and Results. ChemBioEng Rev..

[24] Valechha D., Megarajan S.K., Fakeeha A.H., Al-Fatesh A.S., Labhasetwar N.K. (2017). Effect of SO_2_ on Catalytic CO Oxidation Over Nano-Structured, Mesoporous Au/Ce_1-x_Zr_x_O_2_ Catalysts. Catal. Lett..

[25] Chen D.C., Tang J., Zhang X.X., Cui H., Li Y. (2018). Sulfur dioxide adsorbed on pristine and Au dimer decorated gamma-graphyne: A density functional theory study. Appl. Surf. Sci..

[26] Spezzati G., Benavidez A.D., DeLaRiva A.T., Su Y.Q., Hofmann J.P., Asahina S., Olivier E.J., Neethling J.H., Miller J.T., Datye A.K. (2019). CO oxidation by Pd supported on CeO_2_(100) and CeO_2_(111) facets. Appl. Catal. B Environ..

[27] Bi F.K., Zhang X.D., Xiang S., Wang Y.Y. (2020). Effect of Pd loading on ZrO_2_ support resulting from pyrolysis of UiO-66: Application to CO oxidation. J. Colloid Interface Sci..

[28] Peterson E.J., Delariva A.T., Lin S., Johnson R.S., Guo H., Miller J.T., Kwak J.H., Peden C.H.F., Kiefer B., Allard L.F. (2014). Low-temperature carbon monoxide oxidation catalysed by regenerable atomically dispersed palladium on alumina. Nat. Commun..

[29] Sharma A.K., Mehara P., Das P. (2022). Recent Advances in Supported Bimetallic Pd-Au Catalysts:Development and Applications in Organic Synthesis with Focused Catalytic Action Study. ACS Catal..

[30] He Y., Luan C., Fang Y., Feng X., Peng X., Yang G., Tsubaki N. (2020). Low-temperature direct conversion of methane to methanol over carbon materials supported Pd-Au nanoparticles. Catal. Today.

[31] Zhou Q., Luo S., Zhang M., Liao N. (2022). Selective and efficient hydrogen separation of Pd-Au-Ag ternary alloy membrane. Int. J. Hydrogen Energy.

[32] Bathla A., Pal B. (2020). Superior co-catalytic activity of Pd(core)@Au(shell) nanocatalyst imparted to TiO_2_ for the selective hydrogenation under solar radiations. Sol. Energy.

[33] Zhang J., Alexandrova A.N. (2013). The Golden Crown: A Single Au Atom that Boosts the CO Oxidation Catalyzed by a Palladium Cluster on Titania Surfaces. J. Phys. Chem. Lett..

[34] Wilburn M.S., Epling W.S. (2019). SO_2_ adsorption and desorption characteristics of bimetallic Pd-Pt catalysts: Pd: Pt ratio dependency. Catal. Today.

[35] Kresse G., Furthmuller J. (1996). Efficient Iterative Schemes for ab initio Total-energy Calculations Using a Plane-wave Basis Set. Phys. Rev. B.

[36] Perdew J.P., Burke K., Ernzerhof M. (1996). Generalized Gradient Approximation Made Simple. Phys. Rev. Lett..

[37] Hammer B., Hansen L.B., Norskov J.K. (1999). Improved Adsorption Energetics within Density-functional Theory Using Revised Perdew-Burke-Ernzerhof Functionals. Phys. Rev. B.

[38] Grimme S. (2006). Semiempirical GGA-type Density Functional Constructed with a Long-range Dispersion Correction. J. Comput. Chem..

[39] Yi H.H., Tao T., Zhao S.Z., Yu Q.J., Gao F.Y., Zhou Y.S., Tang X.L. (2021). Promoted adsorption of methyl mercaptan by gamma-Al_2_O_3_ catalyst loaded with Cu/Mn. Environ. Technol. Innov..

[40] Lassoued A., Dkhil B., Gadri A., Ammar S. (2017). Control of the shape and size of iron oxide (alpha-Fe_2_O_3_) nanoparticles synthesized through the chemical precipitation method. Results Phys..

[41] Jayaseelan C., Ramkumar R., Rahuman A.A., Perumal P. (2013). Green synthesis of gold nanoparticles using seed aqueous extract of Abelmoschus esculentus and its antifungal activity. Ind. Crops Prod..

[42] Bukhtiyarov A.V., Prosvirin I.P., Panafidin M.A., Fedorov A.Y., Klyushin A.Y., Knop-Gericke A., Zubavichus Y.V., Bukhtiyarov V.I. (2021). Near-Ambient Pressure XPS and MS Study of CO Oxidation over Model Pd-Au/HOPG Catalysts: The Effect of the Metal Ratio. Nanomaterials.

[43] Qian K., Luo L.F., Jiang Z.Q., Huang W.X. (2017). Alloying Au surface with Pd reduces the intrinsic activity in catalyzing CO oxidation. Catal. Today.

[44] Bukhtiyarov A.V., Prosvirin I.P., Saraev A.A., Klyushin A.Y., Knop-Gericke A., Bukhtiyarov V.I. (2018). In situ formation of the active sites in Pd-Au bimetallic nanocatalysts for CO oxidation: NAP (near ambient pressure) XPS and MS study. Faraday Discuss..

[45] Modelska M., Binczarski M.J., Kaminski Z., Karski S., Kolesinska B., Mierczynski P., Severino C.J., Stanishevsky A., Witonska I.A. (2020). Bimetallic Pd-Au/SiO_2_ Catalysts for Reduction of Furfural in Water. Catalysts.

[46] Huang X.Y., Akdim O., Douthwaite M., Wang K., Zhao L., Lewis R.J., Pattisson S., Daniel I.T., Miedziak P.J., Shaw G. (2022). Au-Pd separation enhances bimetallic catalysis of alcohol oxidation. Nature.

[47] Bukhtiyarov A.V., Prosvirin I.P., Bukhtiyarov V.I. (2016). XPS/STM study of model bimetallic Pd-Au/HOPG catalysts. Appl. Surf. Sci..

[48] Chenakin S.P., Melaet G., Szukiewicz R., Kruse N. (2014). XPS study of the surface chemical state of a Pd/(SiO_2_+TiO_2_) catalyst after methane oxidation and SO_2_ treatment. J. Catal..

[49] Venezia A.M., Murania R., Pantaleo G., Deganello G. (2007). Pd and PdAu on mesoporous silica for methane oxidation: Effect of SO_2_. J. Catal..

[50] Liotta L.F., Di Carlo G., Pantaleo G., Venezia A.M., Deganello G., Borla E.M., Pidria M.F. (2007). Pd/Co_3_O_4_ catalyst for CH_4_ emissions abatement: Study of SO_2_ poisoning effect. Top. Catal..

[51] Spezzati G., Su Y.Q., Hofmann J.P., Benavidez A.D., DeLaRiva A.T., McCabe J., Datye A.K., Hensen E.J.M. (2017). Atomically Dispersed Pd-O Species on CeO(2)(111) as Highly Active Sites for Low-Temperature CO Oxidation. ACS Catal..

[52] Zorn K., Giorgio S., Halwax E., Henry C.R., Gronbeck H., Rupprechter G. (2011). CO Oxidation on Technological Pd-Al_2_O_3_ Catalysts: Oxidation State and Activity. J. Phys. Chem. C.

[53] Celorrio V., Quaino P.M., Santos E., Florez-Montano J., Humphrey J.J.L., Guillen-Villafuerte O., Plana D., Lazaro M.J., Pastor E., Fermin D.J. (2017). Strain Effects on the Oxidation of CO and HCOOH on Au-Pd Core-Shell Nanoparticles. ACS Catal..

[54] Tran-Thuy T.M., Yu T.L., Lin S.D. (2022). How H_2_O may influence ambient CO oxidation over Au/BN. Appl. Catal. B Environ..

[55] Li S., Hasan N., Ma H., Li O.L., Lee B., Jia Y.F., Liu C. (2022). Significantly enhanced photocatalytic activity by surface acid corrosion treatment and Au nanoparticles decoration on the surface of SnFe_2_O_4_ nano-octahedron. Sep. Purif. Technol..

[56] Li Q.Z., Wu C.J., Wang K., Wang X.X., Chen X., Dai W.X., Fu X.Z. (2022). Comparison of the catalytic performance of Au/TiO_2_ prepared by in situ photodeposition and deposition precipitation methods for CO oxidation at room temperature under visible light irradiation. Catal. Sci. Technol..

[57] Martin N.M., Skoglundh M., Smedler G., Raj A., Thompsett D., Velin P., Martinez-Casado F.J., Matej Z., Balmes O., Carlsson P.A. (2017). CO Oxidation and Site Speciation for Alloyed Palladium-Platinum Model Catalysts Studied by in Situ FTIR Spectroscopy. J. Phys. Chem. C.

[58] Jia S.H., Pu G., Xiong W.C., Wang P.C., Gao J., Yuan C. (2021). Investigation on Simultaneous Removal of SO_2_ and NO over a Cu-Fe/TiO_2_ Catalyst Using Vaporized H_2_O_2_: An Analysis on SO_2_ Effect. Ind. Eng. Chem. Res..

[59] Shigenobu S., Sugiyama T., Hojo H., Einaga H. (2022). Enhanced Benzene Oxidation of Sintered Pd/gamma-Al_2_O_3_ Catalysts by SO_2_ Treatment. Catal. Lett..

[60] Ye Y.L., Xie J.L., De F., Wang X.H., He F., Jin Q.Q. (2022). Effect of acid treatment on surfaces of activated carbon supported catalysts for NO and SO_2_ removal. Fuller. Nanotub. Carbon Nanostruct..

[61] Maumau T.R., Modibedi R.M., Mathe M.K. Electro-oxidation of alcohols using carbon supported gold, palladium catalysts in alkaline media. Proceedings of the 1st Africa Energy Materials Conference (AEMC).

[62] Han Y.F., Wang J.H., Kumar D., Yan Z., Goodman D.W. (2005). A kinetic study of vinyl acetate synthesis over Pd-based catalysts: Kinetics of vinyl acetate synthesis over Pd-Au/SiO_2_ and Pd/SiO_2_ catalysts. J. Catal..

[63] Gao F., Wang Y.L., Goodman D.W. (2010). Reaction Kinetics and Polarization-Modulation Infrared Reflection Absorption Spectroscopy (PM-IRAS) Investigation of CO Oxidation over Supported Pd-Au Alloy Catalysts. Phys. Chem. Chem. Phys..

