# Supplementary material for: The Enhancement of CO Oxidation Performance and Stability in SO2 and H2S Environment on Pd-Au/FeOX/Al2O3 Catalysts
Source: Materials (Basel). 2023 May 16;16(10):3755. doi: 10.3390/ma16103755 (PMC10221715; doi:10.3390/ma16103755)
Supplement: Supplementary file 1 [file materials-16-03755-s001.zip › materials-2305649-supplementary.pdf]

Supplementary Materials

Characterization analysis of Pd-Au/FeO_x/Al_2O_3 catalysts with 5 types of Pd: Au mass ratios

Pd-Au/FeO_x/Al_2O_3 catalysts with Pd: Au mass ratios of 1:1, 2:1, 3:1, 5:1, and 10:1 were prepared by adjusting the Pd mass loading while keeping the nanogold particles loading at 2 wt% constantly. Different ratios of Pd-Au/FeO_x/Al_2O_3 catalysts were thus obtained through the preparation methods.

The addition of noble metals as the second active component to Au/FeO_x/Al_2O_3 catalysts can enhance their activity, while the introduction of Pd as the second active component can alter their activity. However, the proportion of Pd-Au bimetallic components can also affect the catalyst's activity. Therefore, to evaluate the effect of the Pd-Au loading ratio (wt%) on the performance of Au/FeO_x/Al_2O_3 catalysts, characterization and evaluation methods, including ICP-OES and CO conversion rate tests were employed for analysis and research.

1.1. Elemental analysis of different Pd- Au/FeO_x/Al_2O_3 catalysts with varying loading ratios

Table S-1 shows that were using ICP-OES to test the 2 wt% Au/FeO_x/Al_2O_3 and Pd-Au/FeO_x/Al_2O_3 catalysts, the nano-Au particle loading was 1.6 wt%, close to the theoretical value of 2 wt%. For the Pd-Au/FeO_x/Al_2O_3 catalyst with a theoretical Au: Pd ratio of 2:1, the nano-Au particle mass loading was 1.7 wt%, and the nano-Pd particle mass loading were 0.7 wt%, with a measured Au: Pd mass ratio close to the theoretical value of 2:1. The actual loading ratios of the other Pd- Au/FeO_x/Al_2O_3 catalysts were also close to the theoretical values. This indicates that both noble metal active components were successfully loaded onto the support. The deviation between the theoretical and actual values may be attributed to incomplete impregnation of the nano-Pd particles with ultrasound assistance and the loss of some nano-gold particles during the preparation by the deposition-precipitation method, which resulted in incomplete deposition on the support.

Table S1. Au and Pd mass loading of catalysts.

Catalyst	Theoretical loading ratio(Au:Pd)	Actual loading value of Au (wt%)	Actual loading value of Pd (wt%)	Actual loading ratio (Au:Pd)
Au/FeO_x/Al_2O_3	\	1.6	\	\
Pd-Au/FeO_x/Al_2O_3	1:1	2.13	1.59	1:1
	2:1	1.66	0.79	2:1
	3:1	1.87	0.53	4:1
	5:1	1.86	0.38	5:1
	10:1	1.83	0.17	11:1

1.2. The CO conversion rates of the Pd-Au/FeO_x/Al_2O_3 catalysts with different loading mass ratios

Based on the activity evaluation of Pd-Au/FeO_x/Al_2O_3 catalysts with different loading ratios, the results are shown in Figure S-1. The catalysts with Au: Pd (wt%) ratios of 1:1, 2:1, 3:1, 5:1, and 10:1 were compared with the 2 wt% Au/FeO_x/Al_2O_3 catalyst with nanoscale gold particle loading. From the figure, it can be seen that only the Pd-Au/FeO_x/Al_2O_3 catalyst with an Au: Pd ratio of 10:1 had a conversion rate below 100% at temperatures below -20℃, while other catalysts achieved complete conversion of 2500 ppm CO in the temperature range of -30℃ to 60℃. This indicates that within the

temperature range of -30℃ to 60℃, the addition of palladium does not reduce the activity of the Au/FeO_x/Al_2O_3 catalyst, and Pd-Au/FeO_x/Al_2O_3 catalysts still maintain complete conversion of CO, except for the catalyst with an Au: Pd (wt%) ratio of 10:1 at -30℃ with a conversion rate of 99.5%.

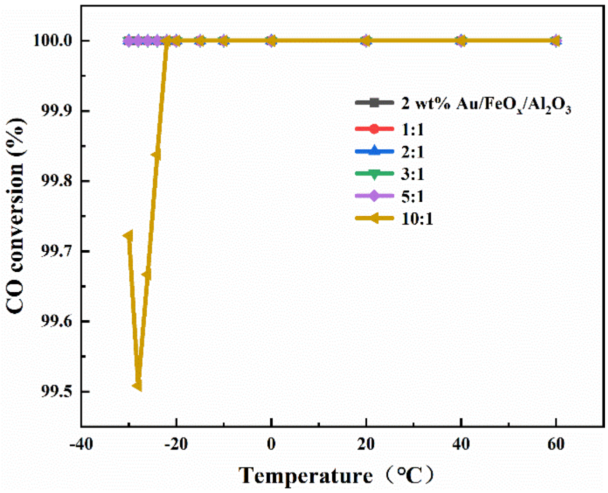

Figure S1. CO conversion of Pd- Au/FeO_x/Al_2O_3 catalysts with different mass loading ratios.

1.3. Conversion of CO to Pd- Au/FeO_x/Al_2O_3 catalysts with different loading ratios after sulphide treatment

To compare the effect of sulfide treatment on the activity of Pd- Au/FeO_x/Al_2O_3 catalysts with different ratios, using CO at a concentration of 2500 ppm to evaluate the catalysts' activity. The results are shown in Figure S-2. The addition of palladium improved the sulfide resistance of the supported gold nanoparticles, and the Au: Pd loading ratio (wt%) significantly affected its sulfide resistance performance. After sulphide treatment, all Pd-Au/FeO_x/Al_2O_3 catalysts achieved complete conversion of CO at 20℃, while the CO catalytic oxidation activity of Au/FeO_x/Al_2O_3 catalysts decreased significantly. The CO catalytic oxidation activity of different catalysts followed this order: 2:1 > 3:1 > 5:1 > 1:1 > 10:1 > 2 wt%. In the range of -30℃ to 60℃, the CO conversion rate of Au/FeO_x/Al_2O_3 catalyst was 23% to 27%. Among Pd-Au/FeO_x/Al_2O_3 catalysts, the 2:1 Au: Pd loading ratio showed the best sulfide resistance performance with a CO conversion rate of 87.2% at -30℃, while the 10:1 mass ratio of Pd-Au/FeO_x/Al_2O_3 catalyst exhibited the weakest sulfide resistance with a CO conversion rate of only 54.8% at -30℃.

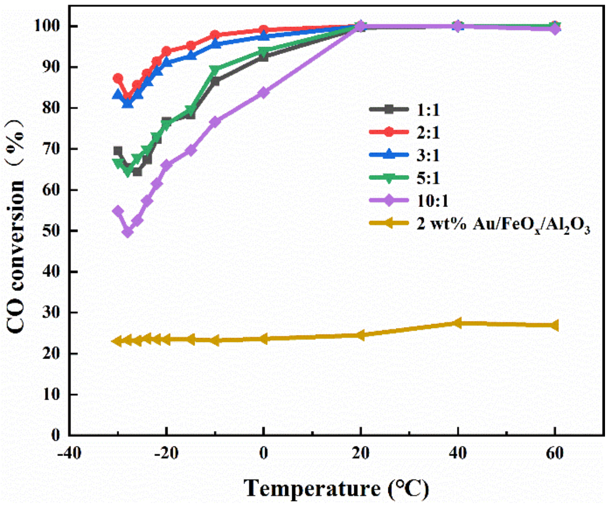

Figure S2. CO conversion of Pd-Au/FeO_x/Al_2O_3 catalysts with different mass loading ratios.after sul-phide pretreatment.

Based on the results obtained from ICP-OES analysis, activity evaluation and the activity evaluation after sulphide pretreatment, it was found that the Pd-Au/FeO_x/Al_2O_3 catalyst with an Au: Pd loading ratio of 2:1 (wt%) exhibited both good catalytic activity for CO oxidation and excellent resistance to sulphide poisoning. Therefore, subsequent catalyst characterization analyses were performed on the Pd-Au/FeO_x/Al_2O_3 catalyst with an Au: Pd loading ratio of 2:1 (wt%) and the Au/FeO_x/Al_2O_3 catalyst with a 2 wt% Au nanoparticle loading, to compare and analyze their CO catalytic oxidation activity, stability, and sulphide resistance.

1. BET analysis

A fully automatic specific area and porosity analyser (BET, 4000E, Quantachrome, USA) was used to determine the specific surface area and pore-size distribution of the carrier and catalyst, the degasification is 300℃, the degasification time is 7 h.

The N_2 adsorption-desorption method measured the specific surface area and pore size distributions of Au/FeO_x/Al_2O_3 and Pd-Au/FeO_x/Al_2O_3 catalysts. The specific surface areas of Au and Pd-Au catalysts were 147.8 and 148.4 m^2/g, respectively. No significant difference was observed between the two catalysts, indicating that the addition of Pd nanoparticles did not change the pore structure of the catalyst. Figure S-3 shows that both catalysts exhibited type-IV adsorption isotherms with similar pore size distributions. These results, combined with the pore size distribution diagram, show that both catalysts are mesoporous.

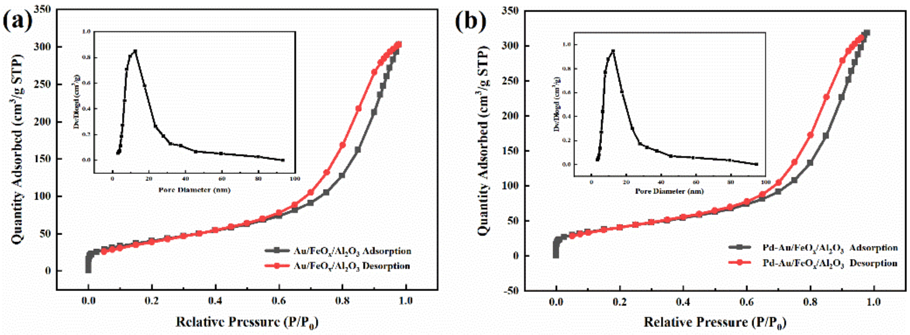

Figure S3. N_2 adsorption-desorption isotherms and pore size distributions of Au/FeO_x/Al_2O_3 (a) and Pd-Au/FeO_x/Al_2O_3 (b).

1. HRTEM of Au/FeO_x/Al_2O_3 and Pd-Au/FeO_x/Al_2O_3 after reaction with CO

As shown in the figure S-4, the particle size distribution range of fresh Au/FeO_x/Al_2O_3 catalyst is 1.5—5 nm, and the particle size distribution range of Au/FeO_x/Al_2O_3 catalyst after CO reaction is larger, becoming 2—8 nm, which indicates that the Co-catalyzed oxidation process causes the aggregation and growth of gold nanoparticles. However, the change of Pd-Au/FeO_x/Al_2O_3 catalyst is not obvious (figure S-5).

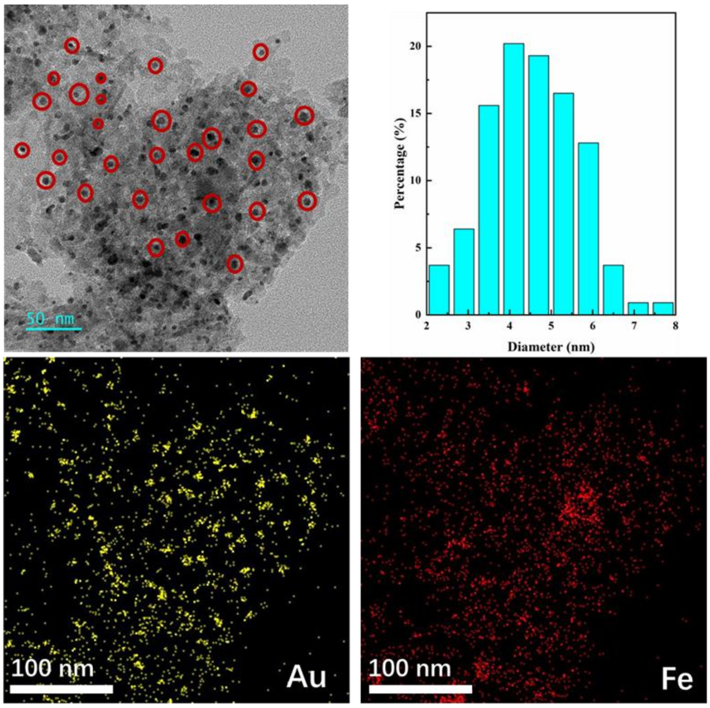

Figure S4. HRTEM of Au/FeO_x/Al_2O_3 catalyst.

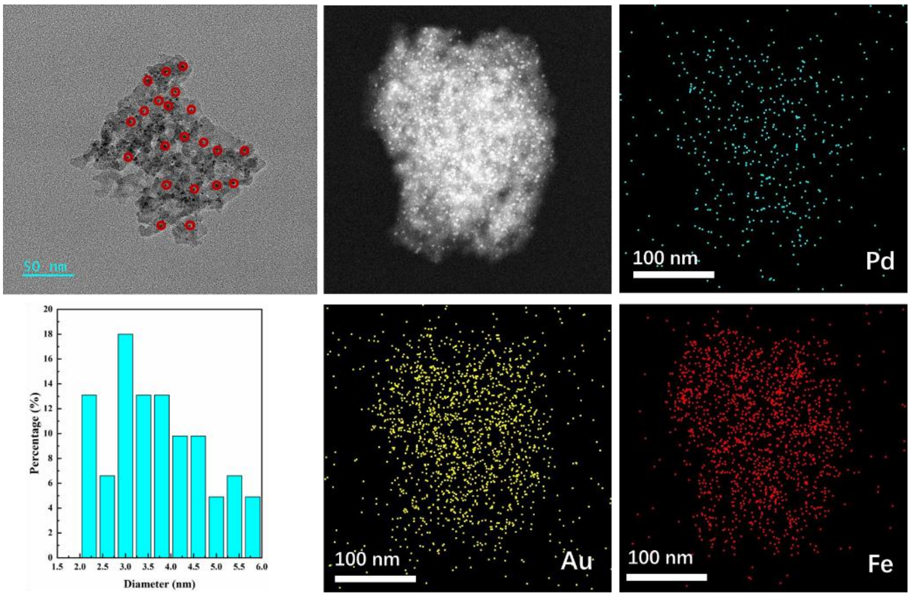

Figure S5. HRTEM of Pd-Au/FeOx/Al2O3 catalyst.
